# Emerging Interaction Patterns in the *Emiliania huxleyi*-EhV System

**DOI:** 10.3390/v9030061

**Published:** 2017-03-22

**Authors:** Eliana Ruiz, Monique Oosterhof, Ruth-Anne Sandaa, Aud Larsen, António Pagarete

**Affiliations:** 1 Department of Biology, University of Bergen, Bergen 5006, Norway; Ruth.Sandaa@uib.no (R.-A.S.); Antonio.Pagarete@uib.no (A.P.); 2 NRL for fish, Shellfish and Crustacean Diseases, Central Veterinary Institute of Wageningen UR, Lelystad 8221 RA, The Nederlands; Monique.oosterhof@wur.nl; 3 Uni Research Environment, Nygårdsgaten 112, Bergen 5008, Norway; Aud.Larsen@uni.no

**Keywords:** *Phycodnaviridae*, Coccolithovirus, Coccolithophore, *Haptophyta*, Killing-the-winner, cost of resistance, infectivity trade-offs, algae virus, marine viral ecology, viral-host interactions

## Abstract

Viruses are thought to be fundamental in driving microbial diversity in the oceanic planktonic realm. That role and associated emerging infection patterns remain particularly elusive for eukaryotic phytoplankton and their viruses. Here we used a vast number of strains from the model system *Emiliania huxleyi*/Emiliania huxleyi Virus to quantify parameters such as growth rate (µ), resistance (R), and viral production (Vp) capacities. Algal and viral abundances were monitored by flow cytometry during 72-h incubation experiments. The results pointed out higher viral production capacity in generalist EhV strains, and the virus-host infection network showed a strong co-evolution pattern between *E. huxleyi* and EhV populations. The existence of a trade-off between resistance and growth capacities was not confirmed.

## 1. Introduction

Since the discovery of high viral concentrations in the marine environment, normally ranging between 10^7^ and 10^11^ virions/L [1], hypotheses regarding the potential impact those viruses could have on their microbial host populations, have been put forward. Viral-induced microbial lysis in Earth’s oceans could amount to an impressive 10^23^ new infections per second, releasing up to 10^9^ tons of cellular carbon every day [2,3]. Consequently, viral lysis contributes greatly to marine biogeochemical cycling of nutrients as well as reducing the transport of organic matter to upper trophic levels in a process known as viral shunt [4,5,6]. Through horizontal gene transfer and the lysis of their hosts, marine viruses contribute to structuring the diversity and composition of microbial communities [7,8,9,10,11].

Viral activity has been suggested as a plausible mechanism contributing to explain Hutchinson’s paradox, which questions the existence of highly diverse planktonic communities in nutrient limited environments [4,12,13]. Viral strain or species-specific lysis may potentially explain the coexistence of cells with different growth and resistance capacities [14,15]. This scenario is contemplated in the Killing-the-Winner (KtW) hypothesis, notably with the concept that resistance has an inherent cost. This trade-off, also known as cost of resistance (COR), ultimately regulate the co-existence of competition specialists (with higher growth rates) and defence specialists (with higher immune capacity against viral infection), respectively [16]. 

COR can be detected by analysing the virus-host infection network patterns (VHINs) that emerge after cross-infectivity experiments [17,18,19]. The most frequently tested VHIN patterns are nestedness and modularity [17,20]. Nested patterns are characterized by specialist viruses tending to infect the most susceptible hosts, while the viruses with broader host-range infect hosts that are more resistant [21]. On the other hand, in modular patterns the interactions tend to occur within different groups of viruses and hosts, but not between groups [17,22]. 

The role of viruses as an important driver of microbial diversity has become clear in prokaryotic-virus systems [23,24,25,26,27] such as the *Pseudoalteromonas* [28] and the *Pseudomonas aeruginosa* host-virus systems, in which resistant cells emerging after infection were less competitive than the sensitive ones [24]. In other prokaryote-virus systems that role remains elusive [29,30,31,32,33,34]. The very few examples of trade-off between resistance and growth rate in eukaryotic hosts include studies on the prasinophyte *Ostreococcus tauri* [35] and the trebouxiophyte *Chlorella variabilis* [36].

Here we aim at getting insight on the main emerging patterns that result from eukaryotic host-virus interactions in the planktonic realm by focusing on *Emiliania huxleyi*, the most abundant and widely distributed calcifying haptophyte in our oceans [37], and its lytic viruses. Mostly known for its impressive blooms [38,39] this microalga is an important player in global geochemical cycles [40,41]. This photosynthetic unicellular eukaryote is infected by *Emiliania huxleyi* viruses (EhV), lytic giant viruses belonging to the genus *Coccolithovirus*, within the *Phycodnaviridae* family. These viruses are ubiquitous in the marine environment [42] and abundant, reaching 10^7^/mL in natural seawater during bloom conditions and from 10^8^ to 10^9^/mL in laboratory cultures [43]. Genomic and metagenomic EhV characterizations show both a global consistency of this viral genome on a planetary scale as well as the maintenance of specific localized genetic traits. For example, despite the high levels of sequence similarity (>95%) between EhV isolates from a Norwegian fjord and the English Channel, these viral populations also contain distinctive genetic traits [44,45,46,47,48,49,50]. It is surprising that these genetic traits have been maintained through decades although no geographical isolation and speciation have occurred to date [45], allowing these viral communities to infect hosts from distant geographic places [44,51]. 

Taking advantage of the large number of *E. huxleyi* cell and EhV lines available for this host-virus system, from diverse geographical origins that include the major oceanic regions, an extensive array of cross-infectivity experiments was conducted in order to investigate parameters such as growth rate (µ), resistance (R), and viral production (Vp). We then confronted possible existence of correlations between those parameters with the theoretical hypotheses (Table 1) that delimit our conception of virus-microbe interactions in the oceans and the way we model those interactions.

## 2. Materials and Methods 

### 2.1. Emiliania Huxleyi and EhV Strains 

Algal strains were obtained from the Roscoff Culture Collection, France; and from the University of Bergen, Norway. A total of 49 *E. huxleyi* strains (Table S1) were maintained in 30 mL polystyrene flasks with IMR ½ medium [69] at 16 °C and a 14:10 h light:dark illumination cycle at 155 µmol photon m^−2^/s irradiance. 

A total number of 13 viral strains were obtained from the Plymouth Marine Laboratory, UK; and from the University of Bergen, Norway (Table S2). For all viral isolates, viral stocks were produced by infection of exponentially growing *E. huxleyi* RCC1257 strain. Viral lysates were centrifuged at 12,000 × g for 20 min and the supernatant was filtered through a 0.45 µm syringe filter (Whatman plc, GE Healthcare Life Sciences, Kent, UK) to remove cellular debris. Viral stocks were kept at 4 °C in the dark and were renewed so often as to never be more than 2 weeks old before inoculation in order to preserve the agent’s viability. Plaque assays were not conducted as haptophytes in general do not grow on agar plates and have only been achieved for a few *E. huxleyi* strains [70,71]. 

### 2.2. Cross-Infectivity Experiments

Cross-infectivity experiments were performed between all the *E. huxleyi* and EhV strains (Table S3). Prior to each experiment, *E. huxleyi* cultures were maintained in exponential growth phase with cell concentrations ranging from 10^5^ to 10^6^ cells/mL. The experiments were performed in 24 well culture plates under the same temperature and light conditions as the general culturing conditions described above. Triplicates of 2 mL of each algal culture (1 × 10^5^ cells/mL) were inoculated with each of the 13 viral strains at a concentration of 1 × 10^6^ viral particles/mL, resulting in a virus to host ratio (VHR) of 10. Three replicates of uninfected culture were also used as a control for each *E. huxleyi* strain. An incubation time of 72 h was chosen because this is consistent with the time scales reported for *E. huxleyi*/EhV selection dynamics observed in the natural environment [72,73,74]. Moreover, preliminary growth tests [75] performed on several *E. huxleyi* strains, did not indicate that prolonged incubation period would contribute essential knowledge on the growth capacity of each strain.

### 2.3. Enumeration of Algae and Viruses 

At times 0 h and 72 h, 500 µL was subsampled from each well to determine algae and virus concentrations using a FACSCalibur BC flow cytometer (Becton–Dickinson, Biosciences, Franklin Lakes, NJ, USA) [76,77,78] provided with an air-cooled laser procuring 15 mW at 488 nm. Viral samples were fixed with 20 µL of glutaraldehyde (25%) for 30 min at 4 °C, and frozen at −80 °C until further use. For flow cytometry analysis, samples were thawed, diluted 500-fold in TE buffer (10:1 mM Tris:EDTA, pH 8, filtered through 0.2 µm), and stained with SYBR Green I 100× diluted (Invitrogen, 1600 Faraday Avenue, PO Box 6482, Carlsbad CA, 92008 United States) for 10 min at 80 °C before analysis. Algal enumeration was conducted on fresh samples, and cell populations were discriminated using chlorophyll auto-fluorescence (670 LP) and SSC signals. Virus populations were determined and enumerated on basis of their green fluorescence (530/30) and SSC signals. 

### 2.4. Growth Rate, Resistance, Viral Production

Growth rates (µ) were calculated for each *E. huxleyi* strain using the control non-inoculated incubations according to the following formula [79]:

µ = Ln (N2/N1)/t
(1)

where N1 and N2 were the cell concentrations at the beginning and end of the experiment, respectively, and t was the incubation time in days. 

The level of resistance of each *E. huxleyi* strain to viral infection was measured in two manners. The first manner (R_1_) was based on the difference of cells that were not lysed after incubation with viruses, compared to the non-inoculated controls. For each *E. huxleyi* strain a resistance value was hence calculated against each of the 13 EhV strains and the 13 resistance values were then averaged to obtain an overall resistance capacity for each alga strain (R_1_). Resistance was also estimated as the number of EhV strains that successfully produced progeny on that host (R_2_).

A value of viral production (Vp), corresponding to the capacity of each viral strain to produce new progeny on a certain host, was calculated for each virus — host pair as the difference between final and initial viral concentrations. These values were averaged to obtain a global infectivity capacity for each viral strain, per algal strain. The maximum amount of viruses that each EhV strain, per algal strain, produced was registered as “Maximum viral production”. 

Potential correlations between the different parameters (growth rate, resistance, and viral production) were investigated with regression slopes and statistical probability analyses, using either Anova (*F*) or Pearson analysis.

A potential impact of domestication on these parameters was also investigated. An analysis was performed on two groups of *E. huxleyi* strains, which were isolated in different periods of time. The periods before and after 2009, respectively, were chosen for an apparent increase in Vp was preliminary observed in strains as old or younger than 2009 (Figure S1).

### 2.5. Host-Virus Network Analysis

In order to test the structure of the infection network, we used the BiMat package for Matlab [21]. This network-based analysis was applied on a binary matrix where 0 referred to no lysis and 1 to lysis. The NODF algorithm was used to measure nestedness and is based on overlap and decreasing fill [80]. It returns a score between 0 and 1, where 1 corresponds to a perfectly nested structure. Modularity (Qb) was calculated using the Leading-Eigenvector algorithm [81]. The value Qb, introduced by Barber [82], is calculated using the standard bipartite modularity function. To quantify the statistical significance of the nestedness (NODF) and modularity (Qb), 100 null random matrices (for each) were created with the null model Equiprobable (a random matrix in which all the interactions are uniformly permuted). 

## 3. Results

Forty-nine *E. huxleyi* strains were characterized according to their ability to grow under a standard set of nutrients, light and temperature conditions. Growth rate (µ) varied significantly among *E. huxleyi* strains, ranging from 0.12 (SD ± 0.01) to 1.11 (SD ± 0.02)/d (registered in strains RCC4533 and RCC1744, respectively) (Figure S2). The difference in growth rate among the algal strains was not related to the ocean they were isolated from (one-way ANOVA *F* (2, 37) = 0.275, *p* = 0. 76). 

**Figure 1:**
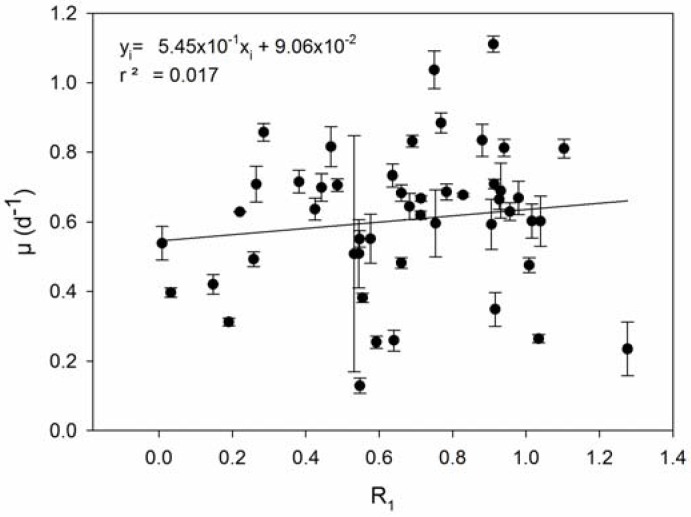
Resistance capacity R_1_ (calculated as the ratio between the number of cells that did not lyse after incubation with viruses and the number of cells in the non-inoculated controls) plotted against growth rate (μ). Error bars show standard deviation (*n* = 3).

**Figure 2:**
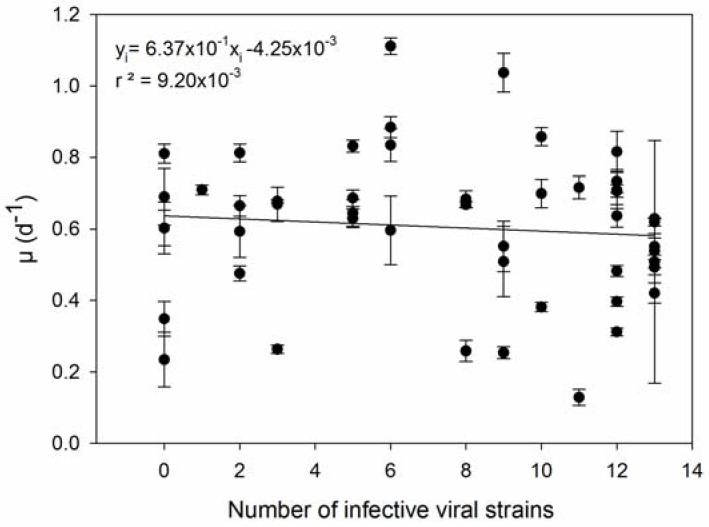
Resistance capacity R_2_ (number of viral strains infecting each algal strain) plotted against growth rate (μ). Error bars show standard deviation (*n* = 3).

**Figure 3:**
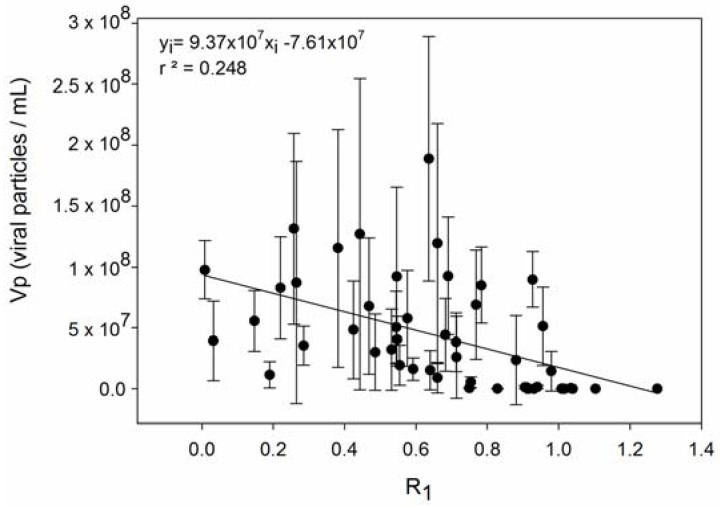
Viral production (Vp) plotted against resistance capacity R_1._ Error bars show standard deviation (*n* = 13).

**Figure 4:**
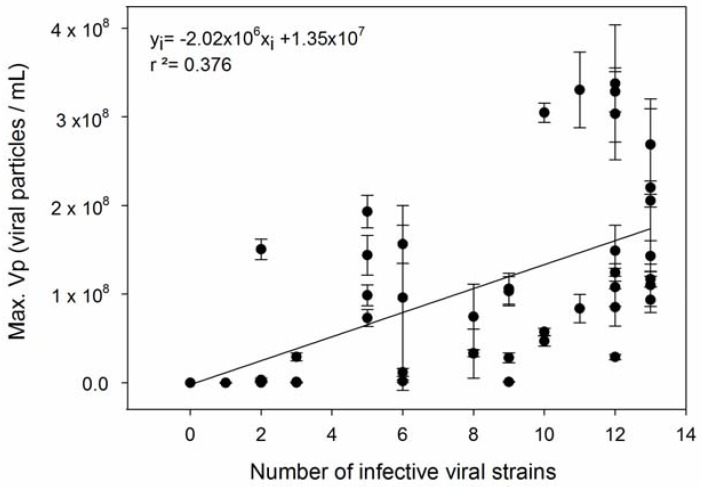
Number of viral strains infecting each algal strain and maximum viral production correlation. Error bars show standard deviation (*n* = 3).

We confronted the observed differences in resistance capacity with the parameters growth rate and viral production, respectively. The level of resistance of *E. huxleyi* to EhV infection was accessed in two manners: (R_1_) percentage of cells that were not lysed after incubation with viruses (Figure S3) and (R_2_) the number of EhV strains that successfully produced progeny on that host, meaning that lower R_2_ levels indicate higher resistance capacity. A trade-off between resistance and growth rate capacities (hypotheses 1 and 3 in Table 1**)** was not confirmed with our results. Neither types of resistance, R_1_ and R_2_, were significantly correlated to growth rate (Pearson’s r = −0.131, *p* = 0.370, and Pearson’s r = −0.0959, *p* = 0.512; respectively) (Figure 1 and Figure 2). R_1_ was indirectly correlated with viral production (Figure 3) (Pearson’s r = −0.499, *p* > 0.01), in accordance with hypothesis 4. R_2_ was significantly and positively correlated with maximum viral production (Pearson’s r = 0.614, *p* < 0.01), which means that the *E. huxleyi* strains that were susceptible to more EhV types were also the ones that presented higher maximum viral production (Figure 4). Viral production and growth rate did not correlate significantly (Pearson’s r = 0.1, *p* = 0.494) (Figure S4) and hence did not confirm hypothesis 2 (Table 1).

Seven out of the 49 *E. huxleyi* strains (RCC1259, RCC1269, RCC3856, 371, P847, PERU15-40 and SO52) were susceptible to infection by all the EhV strains tested, while 6 *E. huxleyi* strains (RCC1211, RCC1218, RCC1235, RCC1256, RCC1276 and RCC3548) were resistant to infection by all the EhV strains tested. When analysing these two groups of *E. huxleyi* strains, no significant differences in growth rate were found (one-way ANOVA F (1, 11) = 0.01592, *p* = 0.90188), while their R_1_ values were significantly different (one-way ANOVA F (1, 11) = 36.8593, *p* = 8.1 × 10^−5^). 

A significant higher viral production was found in the most recently isolated algal strains (one-way ANOVA F (1, 47) = 30.36, *p* = 1.5 × 10^−6^). For the other parameters (growth rate, R_1_ and R_2_) there were no significant differences between younger and older strains (one-way ANOVA F (1, 47) = 1.094, *p* = 0.30; one-way ANOVA F (1, 47) = 0.106, *p* = 0.745; one-way ANOVA F (1, 47) = 0.909, *p* = 0.345; respectively).

**Figure 5:**
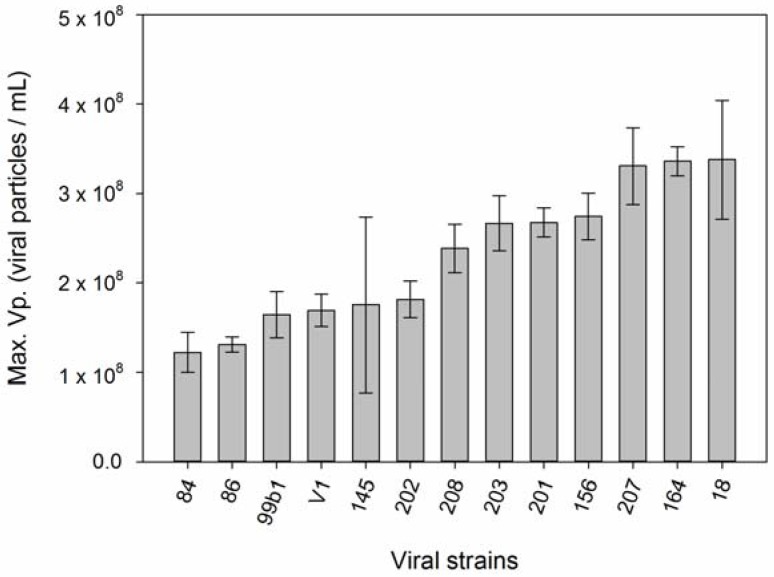
Differences between maximum viral production among EhV strains. Error bars show standard deviation (*n* = 49).

We observed significant variation in “Maximum viral production” capacity among the different EhV strains (Figure 5). Those differences did not translate into significant differences in “Average Viral Production” (Figure S5), as the capacity of each EhV to produce progeny depended very much on which host strain it was infecting. Host-ranges among EhV strains also proved very variable, from generalists that infected up to 36 host strains (e.g., EhV-207) to specialists capable of infecting only 1 strain (e.g., EhV-99b1). Surprisingly, and against the prediction in hypothesis 5, generalist viral strains (EhV-164, EhV-202, EhV-208, EhV-201 and EhV-207) produced significantly more virus progeny viral production (one-way ANOVA *F* (1, 8) = 8.123, *p* = 0.021) than specialist strains (EhV-99b1, EhV-203, EhV-156, EhV-86, and EhV-145).

**Figure 6:**
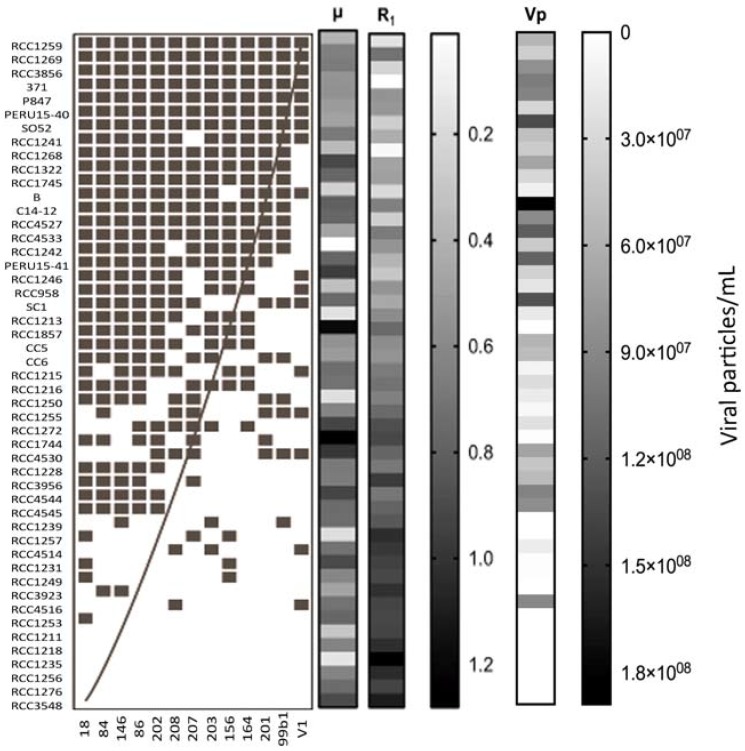
Viral-host infectivity network with a clear nested pattern (NODF value of 0.60) where specialist viruses tend to infect the most susceptible hosts, while viruses with broader host-range infect hosts with higher resistance. ■: infection; □: no infection. Sidebars represent μ, R_1_ and Vp parameters, respectively.

The bipartite network analysis applied to the whole host-range matrix displayed a nested structure (Figure 6) with a NODF value of 0.60. In that nested pattern there was a tendency for hosts with higher resistance to only be infected by more generalist viruses, while specialist viruses tend to infect the most sensitive hosts. 

## 4. Discussion

Since Hutchinson first stated the Paradox of the phytoplankton in the early sixties, many hypotheses explaining the high diversity in the oceans have been postulated [13]. Among these, viral activity has proven to be a potential disrupter on equilibrium in planktonic communities [4,12]. Due to the lack of quantitative data for viral-host interactions, especially in marine micro-eukaryotic organisms, we therefore decided to perform a vast survey on strains of the ubiquitous and environmentally relevant coccolithophorid *Emiliania huxleyi* sp. (*E. huxleyi*) (Lohman) and its virus, *Emiliania huxleyi* virus (EhV), and investigate for emerging patterns resulting from this arms race. 

Among the different hypotheses tested (Table 1) was the existence, or not, of a clear trade-off between resistance and growth rate (COR). COR has been previously confirmed in some bacteria-virus systems [26,27,28,29,30], and is fundamental in the formulation of the Killing the Winner model [19]. In our study we did not observe a clear COR trade-off in the *E. huxleyi*-EhV system. Instead, we found that highly resistant algal strains were capable of growing at high rates. This indicates that, at least in this system, viruses may not be the main selective force acting upon their hosts or, that if they are, their impact is camouflaged by antagonic impacts from other selective factors (e.g., different adaptation to the standard culture conditions used). However, it could be that viral-imposed selection was so strong that it would result in an emerging global cost of resistance observable on *E. huxleyi* strains independently of their inherent local adaptations. An approximation to such global “cost of resistance” is precisely the parameter value used when trying to model the interactions between viruses and their hosts [83]. Its prominence in current models justified the present attempt to evaluate its real extension.

When Avrani and colleagues [29] observed a similar response in viral resistant *Prochlorococcus* strains, they also found that the reduced growth rates increased after 7 months and that these strains reduced their resistance against the viruses [84]. The changes in growth rate and resistance occurred as independent events, indicating that the selection pressure on these phenotypes was decoupled. Decoupled selective pressure for growth rate and resistance may be the reason for the lack of correlation between these parameters in our study as well. 

COR not being observed for the *E. huxleyi*-EhV system using our approach is not necessarily proving it does not exist or that it is irrelevant. As also tried in the current study, COR is often measured as reduction of growth rates in the resistant host [23,24,25,26,27], but other CORs, like altered susceptibility to other viruses and possibly also to some bacteria [85], have also been argued [29,84,86,87,88]. Trade-off might also emerge when strains with different resistance capacities are put under competition for a limited level of nutrients [30,33,89,90], and this is the logical follow up to our study. Another aspect to take into account is the potential impact that domestication has on the isolated strains [91]. In vitro growing conditions (nutrients, light, temperature) are inevitably different from what the cells would be experiencing in the natural environment. Particularly, in vitro cells are released from viral pressure, a situation that, with time, could potentially erase the selective traits that viruses might impose on cells in the natural environment. A sign of domestication-related effects in our case was the lower viral production capacity observed for “older” strains (isolated before 2009). 

Patterns other than COR that shed light on the global interaction between *E. huxleyi* and EhV did, however, emerge in this study. Contrary to our expectations [14,68], we observed a tendency for generalist viruses (e.g., EhV-207) to produce more progeny than the specialists (e.g., EhV-86). It was recently reported that a generalist EhV strain could outcompete a specialist 8 h post infection [92]. One explanation for this apparent difference in infective success between generalist and specialist viruses may thus be a trade-off where high host-range/replication rates are associated with hindered progeny (new virions) fitness [64,93,94,95,96]. An alternative possibility could be the presence of an “un-costly” strong adaptive potential to new hosts, as shown for the Tobacco etch potyvirus (TEV) [97]. It also has to be taken into consideration that viral infective performance; such as viral adsorption coefficient and burst size also depends strongly on host traits. In the current study, a set of *E. huxleyi* strains were the ones that presented the higher viral production, independently of the EhV strain that was infecting them. Such added levels of complexity create niches for different strains of viruses and hosts with different infection and resistance capacities, respectively, to coexist. The patterns emerging from the interaction between *E. huxleyi* and EhV indicate that there’s a plethora of niches that create the possibility for co-existence of viruses and hosts with unexpected trait capacities. Notably, viral strains with narrower host-ranges and smaller virion production competing with generalist strains. Future studies should try to evaluate the possibility of take-over in the case of two specialist or generalist strains. 

The emerging virus-host interaction network (VHIN) pattern showed a significant nestedness match between viral strains and their hosts. A nested structure like this is considered to result from sequential gene-for-gene (GFG) adaptations [98,99]. In the GFG model one genotype replaces another leading to continued fitness improvements of both, host and virus populations, resulting in an everlasting arms race dynamics. Different mesocosm studies on natural *E. huxleyi*/EhV communities [73,74] have shown that host and viral strain diversity can co-change in very short periods of just a few days during *E. huxleyi* blooms. This supports the Arms Race dynamics indicated by our VHIN. Future studies should evaluate the potential for strains with similar host-range capacity to take-over one another. The currently observed cross-infection network did not however have a perfect nested structure. An alternative co-evolution mechanism, termed diffuse co-evolution, appears to be more adequate for multi-species and/or multi-strain communities where selection pressures due to one species, can change in the presence of other species [17,100]. In order to predict diffuse co-evolution, however, experiments in which the different species/strains could interact, allowing real fitness costs associate to both, viruses and hosts, to arise [100] are necessary.

As also previously shown, the same *E. huxleyi* viruses (isolated in the English Channel and the Norwegian fjords) proved able to infect *E. huxleyi* hosts isolated in a large spatio-temporal scale [44,51], indicating a strong genomic consistency between geographically distant EhV strains. Nonetheless, and despite high abundance of conserved genomic sequences among these strains, significant genomic variety is also documented [44,73,101]. As EhVs are enveloped viruses [102], their entry mechanism should be endocytosis or fusion of the envelope with the host’s membrane and the progeny release through a budding mechanism [103]. Such an infection mechanism potentially generates a highly lipid-specific contact between host and virus. The host, *E. huxleyi*, has high phenotypic plasticity [104,105,106,107,108] and adaptation capacity [104,109,110,111,112] that could result in ecotypes that respond differently to viral infection [37,108,112]. Even if genes associated with virus susceptibility have been found within non-core regions of the *E. huxleyi* genome [108], our results did not show significant differences in growth rate, resistance, or viral production in hosts from very distant geographical locations. Hence, despite the recognized genetic variability in both host and virus, our results suggest a globally, non-segregated evolution process between *E. huxleyi* and EhV [113].

In conclusion, and despite a lack of supporting evidence of a trade-off between resistance and growth capacities, our results did indeed, through the nested host-virus interaction pattern, demonstrate a strong co-evolution pattern between *E. huxleyi* and EhV populations. The absence of trade-off between growth rate and resistance, invites us to think that EhVs may not be the main force driving the *E. huxleyi* selection, and that other fitness costs, which passed unnoticeably in the present study, exist. Further work should aim at unravelling these.

## Figures and Tables

**Table 1:** Hypotheses tested in the current study based on outcome of previous virus-host interaction studies. µ: growth rate; R: resistance; Vp: viral production.

Number	Hypothesis	Reference
1	Resistance is associated with reduced growth rates (COR).	Prokaryotes: [23,24,25,27,28,29,52,53,54,55] Eukaryotes: [35,36,56,57]
2	Host strains with higher µ produce more viruses.	[58,59,60,61,62,63,64,65,66]
3	Host strains with higher µ are infected by more viral strains.	[36]
4	Host strains with higher R produce fewer viruses.	[56,67]
5	Specialist viruses have higher Vp than generalists.	[14,68]

## Supplementary Materials

The following are available online at www.mdpi.com/1999-4915/9/3/61/s1, Table S1: *E. huxleyi* strain information, in blank = No information; Table S2: EhV strain information; Table S3: Measured parameters from the cross-infectivity experiments between each *E. huxleyi*-EhV pair. fC = final concentration of *E. huxleyi* cells in cells/mL, µ = *E. huxleyi* growth rate, R_1_ = percentage of cells that were not lysed after incubation with viruses, compared to the non-inoculated controls, Vp = viral production in viral particles/mL, R2 = number of EhV strains that successfully produced progeny on that host, R1 AV = averaged R1 for each algal strain, Vp AV = averaged Vp for each algal strain in viral particles/mL, Max. Vp AV = averaged maximum Vp for each algal strain in viral particles/mL, SD = standard deviation; Figure S1: Correlation between viral production per host cell (Vp) and isolation year of the algal strains. Error bars show standard deviation (*n* = 13); Figure S2: Growth rates (µ/d) measured for control samples of all of the *E. huxleyi* strains (see Table S1 for strain information) used in the infection experiment measured over a period of x days and, calculated according to Levasseur et al. (1993). Values correspond to the control samples. Error bars show standard deviation (*n* = 3); Figure S3: Resistance strategy (R) for each *E. huxleyi* strain. Error bars show standard deviation (*n* = 13); Figure S4: Correlation between growth rate (µ) and viral production per host cell (Vp), in viral particles/mL. Error bars show standard deviation (*n* = 13); Figure S5: Average viral production per EhV strain, for all the algal strains. Error bars show standard deviation (*n* = 49).
