# Correction: Ruiz, E. et al. Emerging Interaction Patterns in the Emiliania Huxleyi-EhV System. *Viruses* 2016, *9*, 61

**DOI:** 10.3390/v9040089

**Published:** 2017-04-24

**Authors:** Eliana Ruiz, Monique Oosterhof, Ruth-Anne Sandaa, Aud Larsen, António Pagarete

**Affiliations:** 1 Department of Biology, University of Bergen, 5006 Bergen, Norway; Monique.oosterhof@wur.nl (M.O.); Ruth.Sandaa@uib.no (R.-A.S.); Aud.Larsen@uni.no (A.L.); Antonio.Pagarete@uib.no (A.P.); 2 NRL for Fish, Shellfish and Crustacean Diseases, Wageningen Bioveterinary Research of Wageningen UR, 8200 AB Lelystad, The Nederlands; 3 Uni Research Environment, Nygårdsgaten 112, 5008 Bergen, Norway

The authors wish to make the following change to their paper [1].

The viral strains in the x axis were not ordered correctly in the original Figure 6. The figure should be replaced with:

The authors apologize for any inconvenience this may cause.

The change does not affect the scientific results. The manuscript will be updated and the original will remain online on the article webpage.

## Figures and Tables

**Figure 6:**
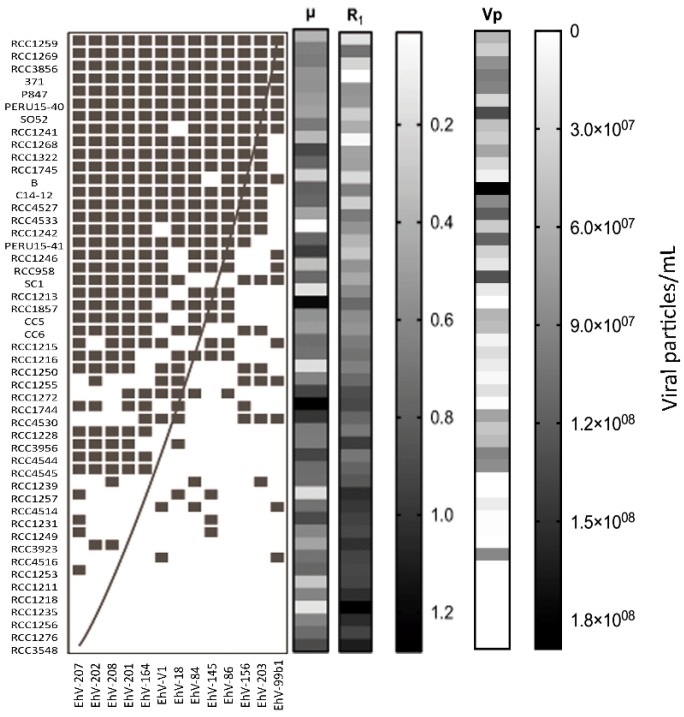
Viral-host infectivity network with a clear nested pattern (NODF value of 0.60) where specialist viruses tend to infect the most susceptible hosts, while viruses with broader host-range infect hosts with higher resistance. ■: infection; □: no infection. Sidebars represent μ, R_1_ and Vp parameters, respectively.
