# Supplementary material for: Emerging Interaction Patterns in the Emiliania huxleyi-EhV System
Source: Viruses. 2017 Mar 22;9(3):61. doi: 10.3390/v9030061 (PMC5371816; doi:10.3390/v9030061)
Supplement: Supplementary file 1 [file viruses-09-00061-s001.zip › supplementary final/TableS1.docx]

**Table S1**. *E. huxleyi* strain information, in blank= No information.

| **Strain** | **Isolation site** | **Coordinates** | **Isolation date** |
| --- | --- | --- | --- |
| RCC1211 | Atlantic Ocean | +35° 41', -7° 33' | 1998 |
| RCC1213 | Mediterranean Sea | 40.68, 14.14 | 2000 |
| RCC1215 | Mediterranean Sea | +41° 40', +2° 48 | 2001 |
| RCC1216 | Pacific Ocean | -42° 18', +169° 50' | 1998 |
| RCC1218 | Pacific Ocean | -42° 18', +169° 50' | 1998 |
| RCC1228 | Atlantic Ocean | +49° 24', -1° 8' | 2003 |
| RCC1231 | Pacific Ocean | -42° 18', +169° 50' | 1998 |
| RCC1235 | Mediterranean Sea | +43° 41', +7° 19' | 2006 |
| RCC1239 | Pacific Ocean | +43° 13', +141° 1' | 2002 |
| RCC1241 | Pacific Ocean | +41° 30', +141° 15' | 2002 |
| RCC1242 | Pacific Ocean | -2° 67', -82° 72' | 1991 |
| RCC1246 | Mediterranean Sea | +41° 36', +2° 39' | 1999 |
| RCC1249 | Mediterranean Sea | +41° 28', +2° 19' | 1998 |
| RCC1250 | Mediterranean Sea | +37° 10', -1° 13' | 1999 |
| RCC1253 | Pacific Ocean | +43° 13', +141° 1' | 2002 |
| RCC1255 | Atlantic Ocean | 59.88, 10.67 | 1905 |
| RCC1256 | Atlantic Ocean | +63° 27', -20° 14' | 1999 |
| RCC1257 | Atlantic Ocean | +63° 27', -20° 14' | 1999 |
| RCC1259 | Atlantic Ocean | +42° 50', -69° 0' | 1990 |
| RCC1268 | Atlantic Ocean | +49° 30', -10° 30' | 2007 |
| RCC1269 | Atlantic Ocean | +49° 30', -10° 30' | 2007 |
| RCC1272 | Atlantic Ocean | +49° 30', -10° 30' | 2007 |
| RCC1276 | Atlantic Ocean | +50° 30', -10° 30' | 2007 |
| RCC1322 | Mediterranean Sea | +36° 15', -1° 35' | 1998 |
| RCC1744 | Atlantic Ocean | +48° 45', -3° 57' | 2007 |
| RCC1745 | Atlantic Ocean | +48° 45', -3° 57' | 2007 |
| RCC1857 | Mediterranean Sea | +34° 8', +18° 27' | 2008 |
| RCC3548 |  |  |  |
| RCC3856 | Pacific Ocean | -30° 15', -71° 42' | 2011 |
| RCC3923 | Pacific Ocean | -36° 39', -73° 20' | 2011 |
| RCC3956 | Pacific Ocean | -30° 15', -71° 42 | 2011 |
| 371 | Atlantic Ocean | 32° N  62° W | 1988 |
| B |  |  | 1991 |
| C14-12 |  |  |  |
| CC5 |  |  |  |
| CC6 |  |  |  |
| P847 |  |  |  |
| PERU15-40 | Pacific Ocean |  | 2015 |
| PERU15-41 | Pacific Ocean |  | 2015 |
| RCC4514 | Atlantic Ocean | +60° 16', +5° 12' | 2009 |
| RCC4516 | Atlantic Ocean | +60° 16', +5° 12' | 2009 |
| RCC4527 | Atlantic Ocean | 38.080884, -26.254634 | 2010 |
| RCC4530 | Atlantic Ocean | 38.080884, -26.254634 | 2010 |
| RCC4533 | Atlantic Ocean | 38.035215, -25.462284, | 2010 |
| RCC4544 | Atlantic Ocean | +27° 59', -15° 22' | 2014 |
| RCC4545 | Atlantic Ocean | +27° 59', -15° 22' | 2014 |
| RCC958 | Pacific Ocean | -8° 20', -141° 15' | 2004 |
| SC1 |  |  |  |
| SO52 |  |  |  |

© 2017 by the authors. Submitted for possible open access publication under the
terms and conditions of the Creative Commons Attribution (CC BY) license (http://creativecommons.org/licenses/by/4.0/).
