# Supplementary material for: Emerging Interaction Patterns in the Emiliania huxleyi-EhV System
Source: Viruses. 2017 Mar 22;9(3):61. doi: 10.3390/v9030061 (PMC5371816; doi:10.3390/v9030061)
Supplement: Supplementary file 1 [file viruses-09-00061-s001.zip › supplementary final/TableS2.docx]

**Table S2.** EhV strain information.

| **Viral isolate** | **Isolation site** | **Isolation date** | **Geographical coordinates** | **Host strain used for viral propagation** |
| --- | --- | --- | --- | --- |
| EhV-18 | English channel | 2008 | 50°15′N/04°13′W | RCC1259 |
| EhV-84 | English channel | 1999 | 50°15′N/04°13′W | RCC1259 |
| EhV-86 | English channel | 1999 | 50°13,79′N/04°9,59′W | RCC1259 |
| EhV-145 | Lossiemouth, Scottland | 2008 | 57°72′N/03°29′W | RCC1259 |
| EhV-156 | English channel | 2009 | 50°15′N/04°13′W | RCC1259 |
| EhV-164 | Scottish shore of Fife | 2008 | 56°26′N/02°63′W | RCC1259 |
| EhV-201 | English channel | 2001 | 50°15′N/04°13′W | RCC1259 |
| EhV-202 | English channel | 2001 | 50°15′N/04°13′W | RCC1259 |
| EhV-203 | English channel | 2001 | 50°15′N/04°13′W | RCC1259 |
| EhV-207 | English channel | 2001 | 50°15′N/04°13′W | RCC1259 |
| EhV-208 | English channel | 2001 | 50°15′N/04°13′W | RCC1259 |
| EhV-99b1 | Raunfjorden, Norway | 1999 | 60.2° N/5.2° E | RCC1259 |
| EhV-V1 | Raunfjorden, Norway | 2003 | 60.2° N/5.2° E | RCC1259 |

© 2017 by the authors. Submitted for possible open access publication under the
terms and conditions of the Creative Commons Attribution (CC BY) license (http://creativecommons.org/licenses/by/4.0/).
